# To the Operating Room! Positive Effects of a Healthcare Clown Intervention on Children Undergoing Surgery

**DOI:** 10.3389/fpubh.2021.653884

**Published:** 2021-04-20

**Authors:** Gabriela Markova, Lukas Houdek, Zuzana Kocabova

**Affiliations:** ^1^Department of Developmental and Educational Psychology, Faculty of Psychology, University of Vienna, Vienna, Austria; ^2^Zdravotni Klaun, Prague, Czechia; ^3^Department of Clinical Psychology, Motol University Hospital, Prague, Czechia

**Keywords:** humor, healthcare clowns, pediatric surgery, parent-child relationship, positive emotions, well-being

## Abstract

In the present study we examined the positive effects of a healthcare clown intervention on children undergoing surgeries, and the role parent-child relationships may play in their effectiveness. Children between 5 and 12 years, who were scheduled to undergo elective surgical procedures in a large university hospital, were randomly assigned to an intervention group (IG; *n* = 35) that was visited by a healthcare clown, and a control group (CG; *n* = 27) that received treatment as usual (i.e., company of parents before the surgery). Children in both groups were videotaped and the videos were later used to rate their activity, arousal, emotional expressivity, and vocalizations. Additionally, children and parents rated their mood and perceived quality of life at several points during the procedure, and parents reported their perceptions of the relationship with their children. Results showed that children in the IG showed more positive emotions and vocalizations than children in the CG. Parents of children in the IG also reported more positive mood than parents of children in the CG. In contrast, children in the CG reported higher quality of life than children in the IG. Importantly, analyses showed considerable effects of the parent-child relationship on all outcome measures. Results of the present study demonstrate that a healthcare clown intervention had some positive effects on behaviors and mood of hospitalized children and their parents. Importantly, our findings also suggest that we need to consider the pre-existing “relationship microcosmos” that the clowns enter when assessing their effectiveness in the hospital.

## Introduction

Hospitals are scary places, especially for children. The fear, anxiety and distress caused by medical treatments increase children's sense of pain and not only disrupt the procedures, but may also have long-term negative emotional and psychological consequences (1). Moreover, an estimated 60% of children suffer from preoperative anxiety (2) which has been identified as an important indicator of postoperative difficulties that can last up to 6 months after the procedure (3). The use of alternative non-pharmacological interventions to manage psychological distress has increased considerably especially in pediatrics (4), including behavioral preparation programs, acupuncture, and art, music, and pet therapies (5, 6). Among these, healthcare clown interventions stand out, because the goal of clowns is not only to distract children from the hospital situation, but to actively evoke positive feelings and increase well-being (7). In fact, already in ancient Greece, doctors were aware of the positive effects of humor on health (8), and theatrical interventions and humor in the treatment of patients have been documented as early as in the thirteenth century [cf. (9)]. Recent research also seems to support the mission of healthcare clown organizations by showing that being exposed to clown antics was associated with subcortical regions involved in experiencing positive reward (10). Surprisingly, the majority of studies examining the effects of a healthcare clown intervention focused on the reduction in negative outcome measures, such as anxiety and negative emotionality, while only few studies have investigated the hypothesized increase in positive outcomes. Moreover, clowns do not exist in a vacuum and when they enter a hospital room, they are incorporated into a pre-existing system that is determined by factors such as the medical conditions of the patients and associated therapeutic procedures, but also the support from the medical team and family functioning (7, 11). For young children, the relationship with their caregivers is a particularly crucial mechanism influencing their regulatory abilities [e.g., (12)], and thus could co-determine whether they experience and show positive emotions in stressful and emotionally arousing situations. Consequently, the aims of the present study were two-fold. On the one hand, we examined whether a healthcare clown intervention evoked positive emotions and well-being in children undergoing surgeries. On the other hand, we specifically investigated the role children's relationship with their caregivers may play in the effectiveness of the clown intervention.

In recent years, it has been increasingly argued that humor and laughter have unique properties that help in coping with pain and stress (8). Previous research has examined the effects of healthcare clown interventions on decreasing anxiety and negative emotions in children during the preoperative period and various, often painful, medical procedures [see (7), for review]. For example, studies have consistently found a reduction in preoperative anxiety measured through observation or self-assessment in children between 3 and 12 years of age (9, 13–18). Others have additionally shown that the clown intervention diminished children's pain perception (19–22), and reduced postoperative maladaptive behavior (20, 23), as well as cortisol (21). A recently published study found that healthcare clown interventions can even reduce the costs associated with hospitalization [i.e., operating room costs, personnel costs; (24)], likely by shortening children's recovery period (25) and reducing the need to use painkillers and sedatives (26). Interestingly, clown interventions also reduce reported situational anxiety in caregivers (9, 13, 16–18, 22, 27, 28). Thus, healthcare clowns seem to effectively decrease anxiety, pain and stress experienced by children and their parents in a preoperative context [see also (7, 9); for systematic reviews].

During their performance, healthcare clowns entertain the goal to improve the emotional state of patients (13). This means that clown interventions not only need to achieve a reduction in negative aspects of the hospital experience, but also evoke positive emotions to create a pleasant atmosphere (7). Although there are arguments that clowns improve children's cooperation during a procedure or examination by creating increasing positive emotions [e.g., (29–31)], only a few studies have investigated whether clowns make hospital patients feel better.

With adult patients, only one study has thus far investigated the effects of healthcare clowns in terms of triggering positive emotions (32). In comparison to a nurse intervention, the authors found that clowns trigger more positive emotions (e.g., fun, entertainment, appreciation) than nurses not only in patients themselves, but also in observers of the clown game [e.g., relatives, friends, parents; (32)]. With pediatric patients, few studies have directly measured or reported positive emotions in their results. Using the Self Assessment Mannequin [SAM; (33)] to measure valence and arousal of emotional states, Fernandes and Arriaga (28) found that children who received a clown intervention reported higher positive affect than those in the control group, but this was regardless of assessment time (i.e., pre- or post-surgical procedure). In contrast, Scheel et al. (34) found no difference between their intervention and control group on emotional valence of the SAM, but instead found an increase in children's salivary oxytocin levels from before hospitalization to after the clown intervention. Because oxytocin may be involved in an allostatic function to counteract experienced stress (35), these results suggest that children who interacted with clowns might have been more at ease. Saliba et al. (21) used the visual analog scale to estimate children's satisfaction with the clown intervention ranging from “very sad” to “really happy” and found that satisfaction with clowns increased from before to after the intervention. However, in their study not all children underwent surgical procedures and the authors did not include an actual control group, thus, together with the only indirect assessment of children's emotions, these results remain difficult to interpret. In a study with 3–7-year-old children, Yildirim et al. (36) found that children who were accompanied by a clown-nurse were more active, had better mood, better communication, and interaction with their parents, and showed better compliance during a burn dressing change compared to a control group. Even though findings of the above-mentioned studies showing an increase in positive emotions are inconsistent and not always specific for the pre-operative context, they suggest that a clown intervention not only reduces children's worries and fears, but may also have a positive influence on emotional experiences of children and their relatives. Moreover, Pinquart et al. (29) reported an improvement in self- and parent-reported psychological well-being in 6–14-year-old patients who received a healthcare clown visit. Although Pinquart et al. (29) did not find a continuation of this effect at a follow-up assessment 4 h later, these results suggest that clowns positively influence, albeit short-term, the well-being of hospitalized children.

To summarize, there is some evidence that clowns can trigger positive emotions and improve well-being in pediatric patients. However, experiencing and appreciating humor in a strange hospital context might be difficult especially for younger children. While several mechanisms have been argued to influence humor perception and understanding [e.g., superiority, relief, see e.g., (37), for an overview], theorists agree that humor involves detecting the discrepancy between expectations and reality [i.e., incongruity; (38, 39)]. An important factor in perceiving incongruity as something amusing is that the incongruous event is considered as non-threatening (37). Accordingly, the context in which the incongruous element occurs determines whether it is interpreted as funny or absurd (38, 40). A playful and non-threatening context is thus a key element to accept an incongruous event as humorous (38, 41). Considering that the experience of humor is based on walking this thin line between what is perceived as threatening vs. funny and the visit of healthcare clowns is an unfamiliar situation in a strange and frightening place, parents may be a particularly helpful source of information regarding how to interpret this ambiguous situation. While many previous studies have observed both children and their caregivers, the effects of the clown intervention were evaluated separately for them. No studies have, thus far, examined the effects of healthcare clowns on pediatric patients as a function of children's relationship with their parents.

Overall, parents could influence the effects of the clowns in that they appreciate the value the clowns bring to the hospital and communicate this appreciation in their stance toward them in the clown situation. Humor, in general, is more engaging with another person, thus parents who laugh with their children could encourage their humor perception and appreciation (42, 43). For example, Mireault et al. (44) showed that already in their first year of life children laughed at absurd events independently of parental affect, but their displays of positive affect were significantly longer when their parents provided them with an affectional cue. It is thus not surprising that humorous interactions are considered a “microcosmos of secure attachment dynamics” [(45), p. 800]. Children are evolutionarily predisposed to turn to their parents in uncertain situations (46–48). The more uncertain a situation is, the more often children look at their caregivers for orientation and interpretation (49), and this referencing back with the caregiver's emotions may be dependent on their overall relationship. As sensitive parents perceive the signals of their children, interpret them correctly, and react promptly and appropriately to their needs (50, 51), children develop a confidence in their caregivers as a reliable source of information about the environment. This not only allows them to indulge in playful exploration of the environment in non-threatening situations, but also rely on parental signals for interpreting an ambiguous situation as harmless and possibly as humorous.

However, the effectiveness of the clown intervention does not merely rely on children's perception of humor that may serve as a distraction, but beyond that on whether humor prompts children's own emotion regulation strategies [see also (52)]. Previous research showed that parenting strongly influences children's emotion regulation strategies [e.g., (12, 53, 54)]. On the one hand, it has been suggested that caregivers' affective behavior mirrors their own emotion regulation capacities, and thus they model different modes of emotion regulation to their children (55–59). On the other hand, children's emotion regulation skills also depend on specific qualities of the relationship with their caregivers. On a broad scale, attachment has been shown to have a large impact on children's emotional development, with securely attached children showing more adaptive emotion regulation strategies [e.g., (60–62)]. More specifically, caregiving qualities such as involvement, warmth, positive responsiveness, and sensitivity have been associated with children emotion regulation [e.g., (63–70)]. By realizing that their signals are correctly interpreted and responded to, children become aware of their influence on the environment mediated through the expressions of their own emotions (71). Thus, emotion regulation is a relational phenomenon where children's experience of self-efficacy can make the feeling of negative emotions less threatening as children feel more capable in dealing with them, possibly via interactions with healthcare clowns.

While prompt and sensitive reactions of parents may determine the extent to which children succeed in orienting themselves to their parents in incongruent situations, negative aspects of a relationship between children and caregivers, such as conflicts and dependence, may be factors that not only lower the trust that children have in their parents, but could also impede an active engagement with the clowns. Although research supporting this argument is scarce, McDowell et al. (67) showed that children who exhibited stronger negative reactions to emotional vignettes were more likely to have parents with more negative relationship qualities. Specifically, parental representations in relating with the child is not only a key feature of their caregiving behavior, but also affects children's emotional and social development (72). Thus, negative parent-child relationship aspects could lead to more negative emotion regulation strategies, and in consequence a rejection of clown humor.

The mission of clowns in a hospital setting is to bring humor and laughter to pediatric patients, and, consequently, their work includes not only the reduction of negative emotions, but also an increase in positive emotions and improvement in mood. However, very few studies have examined whether the clown intervention actually elicits positive emotions and increases well-being in hospitalized children and their parents. Moreover, young children's appreciation of humor in a stressful context, such as the hospital setting, is likely dependent on their inclination to be engaged with the clowns and this, in turn, may be a function of the relationship with their caregivers. Consequently, in the present study we investigated the positive effects of a healthcare clown intervention (i.e., decrease in negative emotions, increase in positive emotions) on children before surgeries and their caregivers, and examined the role of caregiver-child relationship in this effect. Children were observed during a sequence of situations before their scheduled elective surgeries in the company of either a clown and their parent (i.e., intervention group) or their parent only (i.e., control group). We hypothesized that children and parents in the intervention group will report more positive mood and well-being than children and parents in the control group. Moreover, we expected that positive child-parent relationship aspects will increase the effectiveness of the healthcare clown intervention.

## Method

### Participants

A total of 62 children between 5 and 12 years of age, who were scheduled for elective surgeries in a large city hospital, and their caregivers participated in the present study. Children and their caregivers were recruited during the surgery consultation 1 day before their scheduled surgery in the Department of Pediatric Surgery, Motol University Hospital in Prague (Czechia). Participating children were scheduled for the following surgical procedures/reasons: hernias (inguinal, umbilical, supraumbilical, hydroceles), circumcision (phimosis), frenulum breve, testicular retention, cystoscopy, small amendment or enlargement of the urethra, hypospadias, scar repairs, removal of minor cutaneous and subcutaneous formations, planned appendectomy, cholecystectomy, or gastroscopy. Caregivers were informed about the goal and methods of the study and if they agreed to participate, then they signed a written informed consent. Additionally, verbal assent was also sought from the children.

Children were randomly assigned to an intervention group (IG; *n* = 35, *M*_age_ = 8.42 years, *SD*_age_ = 2.07, 9 female) and a control group (CG; *n* = 27, *M*_age_ = 8.31 years, *SD*_age_ = 1.98, 11 female). Table 1 shows the background characteristics of children and their parents for the whole sample as well as the two groups separately. The groups did not differ in age (*p* = 0.553), nor on any of the background characteristics. Moreover, we assessed whether children had previous experience with clowns in and outside the hospital (see Table 1) and found no difference between the groups on this variable (*p* = 0.884).

**Table 1:** Descriptive statistics for the background characteristics of the tested sample.

	**M (SD)**		
	**Total sample**	**IG**	**CG**
	***N* = 62**	***n* = 35**	***n* = 27**
Child age (years)	8.03 (2.32)	8.00 (2.17)	8.08 (2.55)
Siblings (count)	1.34 (.98)	1.23 (.81)	1.48 (1.16)
Maternal age (years)	38.37 (5.25)	37.29 (4.71)	39.77 (4.22)
Paternal age (years)	40.78 (6.04)	39.35 (6.72)	42.72 (6.07)
	**Frequency (%)**		
	**Total sample**	**IG**	**CG**
	***N*** **= 62**	***n*** **= 35**	***n*** **= 27**
Child gender (female)	20 (32.26)	9 (25.71)	11 (40.74)
Nationality (CZ)	58 (93.55)	31 (88.57)	27 (100)
Maternal education (university)	18 (29.03)	12 (34.29)	6 (22.22)
Paternal education (university)	18 (29.03)	11 (31.43)	7 (25.93)
Child experience with HC clowns	13 (20.97)	8 (22.86)	5 (18.52)

### Procedure

**Figure 1:**
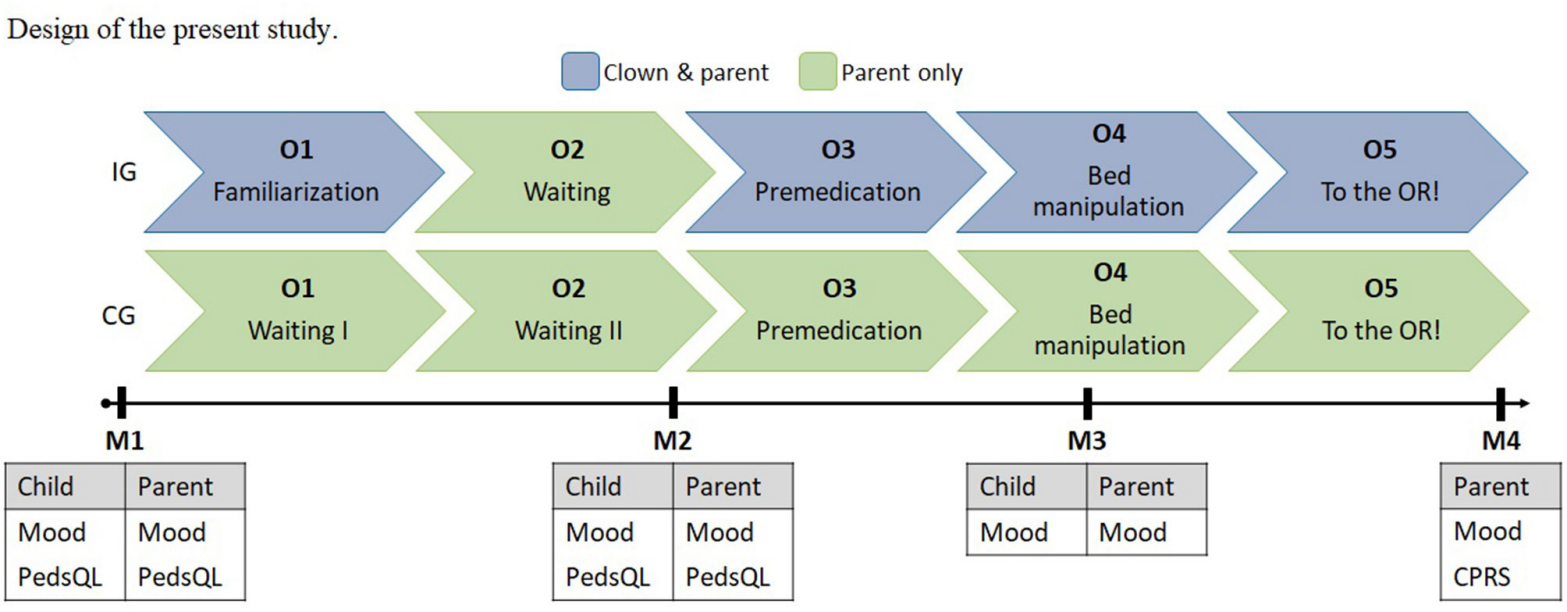
Design of the present study.

Figure 1 shows the design of the present study. Children were observed during five observation situations: (O1) familiarization/waiting; (O2) waiting; (O3) premedication; (O4) bed manipulation; and (O5) on the way to the operating room.

In the intervention group, children were visited by an individual healthcare clown as part of the regular clown program in the hospital, which specifically aims at accompanying children and their parents to surgeries. Eight different clowns, who were members of a professional healthcare clown organization, were involved in the present study. All clowns were professional artists who have undergone extensive training within their organization (i.e., artistic workshops, seminars on healthcare, sociology, psychology, education etc.). Only clowns with at least 5 years of experience are selected by the artistic management of the clown organization for this program, and clowns additionally receive special training for their visits to the surgical ward. Clowns first visited children during O1, where they introduced themselves and warmed up with the children and caregivers. During O2, no clowns were present and children and their caregivers were left alone. Clowns may have visited the families at some point during this phase, but this was not included in the observation. Clowns then returned when children were about to receive premedication (i.e., sedative drops; O3), and then stayed with and accompanied children until before they were moved to the operating room (O4 and O5). Clowns were not present during anesthesia induction. Clowns performed verbal and physical improvisation within the framework of their individual clown characters, including music, magic tricks, etc.

In the control group, children received treatment as usual and were observed in the company of their caregivers and medical staff. While O1 and O2 were the same for the control group and consisted of the families waiting for procedures to commence, all other phases were identical as for the intervention group.

The mean observation duration of each phase in the respective group are displayed in Table 2. While the observation time of O1, O2, O3, and O5 did not differ between the two groups (*p*s = 0.740–0.087), O4 was significantly longer in CG than in IG, *t*_(37)_ = 2.527, *p* = 0.016.

**Table 2:** Mean (SD) observation duration (in min) of each phase for the respective group.

	**O1**	**O2**	**O3**	**O4**	**O5**
IG	5.94 (2.76)	4.66 (1.83)	8.37 (6.09)	0.49 (0.20)	1.63 (0.61)
CG	4.85 (1.87)	4.49 (1.78)	10.07 (8.83)	0.74 (0.39)	1.44 (0.41)

### Measures

As displayed in Figure 1, there were five observation points (O) and four measurement points (M). During the observation points, we used the Modified Yale Preoperative Anxiety Scale – short version [mYPAS; (73)] to rate children's behavior.

#### mYPAS

All observation points were recorded with a digital camera, and these recordings were then used to rate children's behaviors on four aspects: activity, emotional expressivity, state of apparent arousal, and vocalizations. Children's behaviors were rated on a 4-point scale for activity, emotional expressivity, and state of apparent arousal, and on a 6-point scale for vocalizations, where lower scores correspond to more positive reactions, and higher scores corresponded to the most negative reactions. Table 3 provides operationalizations for each rating of the four behaviors coded. Ratings were given for every minute of each observation phase and then a mean score was computed for each phase to account for variations in observation duration, with lower scores suggesting more positive children's behaviors. Inter-rater reliability between two independent observers was high for all behavioral categories of the mYPAS, ranging from κ = 0.86–0.91.

**Table 3:** The mYPAS scoring criteria.

Activity
	1	Moves toward the clown/parent, initiates interaction/play with clowns
	2	Not exploring or playing with clowns/parents, looks around or away
	3	Shows no interest, unfocused, frenetic/frenzied movement or play
	4	Squirming, pushes away, actively trying to get away
State of apparent arousal
	1	Alert, looks around occasionally, notices what clown/parent/staff does
	2	Withdrawn, sitting still and quiet
	3	Vigilant, looking around quickly, may startle, eyes wide, body tense
	4	Panicked whimpering, may be crying or pushing others away, turns away
Emotional expressivity
	1	Apparently happy, smiling, or concentrating on play
	2	Neutral, with no visible expression on face
	3	Worried and/or frightened, sad, or tearful eyes
	4	Distressed, crying, extremely upset
Vocalizations
	1	Asking and answering questions, commenting, babbling, laughing,
	2	Whispering, nodding
	3	Quiet, no sounds or responses to adults
	4	Whimpering, moaning, groaning, silently crying
	5	Crying or may be screaming “no”
	6	Crying, screaming loudly

During the measurement points, various self-rating scales and questionnaires were administered to children and their caregivers as specified in Figure 1.

#### Mood Rating

**Figure 2:**
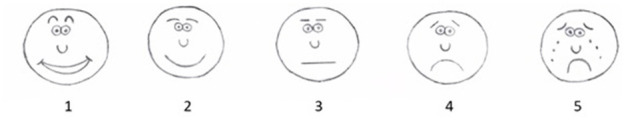
Mood rating scale.

The mood of children and caregivers was assessed on a 5-point rating scale using faces (see Figure 2). Analogous to the mYPAS, low scores correspond to more positive mood ratings.

#### Quality of Life

Health-related quality of life (HRQL) in children was assessed using the Pediatric Quality of Life Inventory [PedsQL; (74)]. The PedsQL is a brief 23-item measure capturing health-related quality of life on four multidimensional scales: physical functioning, emotional functioning, social functioning, and school functioning. These scales were combined to summary scores reflecting physical, psychosocial and total health-related quality of life (range = 0–100 for each summary score), with high scores indicating higher quality of life. The PedsQL was administered to children to measure their own assessment of their own quality of life (i.e., self-report), as well as to caregivers to assess the caregivers' assessment of their children's quality of life (i.e., proxy-report). Internal consistency of the PedsQL showed moderate reliability for the child self-report and parental proxy-report at both measurement points, ranging from α = 0.49–0.72.

#### Caregiving Relationship

Caregivers were administered the Child-Parent Relationship Scale [CPRS; (72)], which is a self-descriptive inventory of parental perceptions of the relationship with their children in the age range of 3–12 years. The questionnaire consists of 30 items which are rated on a 5-point Likert scale. The scores of these items were summed to form three subscales: conflict (range = 12–60), closeness (range = 10–50), and dependence (range = 4–20). In the examined sample, 88% of mothers completed this questionnaire. Internal consistency of the CPRS showed moderate reliability with α = 0.62 for conflict, α = 0.53 for closeness, and α = 0.53 for dependence.

### Statistical Analyses

All statistical analyses were conducted in RStudio (75). To examine whether the healthcare clown intervention had an effect on children's and parental behavior and emotions, we calculated separate linear mixed-effects models [package *lme4*; (76)] for each of the following outcome variables: mYPAS aspects, mood ratings, PedsQL. Children's and parental reports were also analyzed separately. In all models, group (intervention, control), time (nominal levels depending on measure), children's age, and CPRS scores for the three subscales (conflict, closeness, dependence) were included as fixed-effects variables and individual children as a random coefficient. *Post-hoc* effects were Bonferroni corrected. Pearson correlations were computed to examine relationships between the utilized measures for each group separately.

## Results

Descriptive statistics for all outcome measures are provided in Tables 4–6. There were no significant differences between the two groups on all CPRS scores (*p*s = 0.299–0.467), suggesting that the two groups were comparable with respect to their reported relationship qualities.

**Table 4:** M (SD) of the mYPAS measures at the respective observation points.

	**O1**	**O2**	**O3**	**O4**	**O5**
**Activity**					
IG	1.030 (0.117)	1.064 (0.164)	1.100 (0.273)	1.250 (0.444)	1.261 (0.423)
CG	1.083 (0.204)	1.047 (0.138)	1.253 (0.423)	1.267 (0.458)	1.333 (0.456)
**State of apparent arousal**					
IG	1.048 (0.156)	1.074 (0.198)	1.112 (0.292)	1.300 (0.470)	1.280 (0.511)
CG	1.097 (0.243)	1.093 (0.242)	1.212 (0.409)	1.267 (0.458)	1.527 (0.700)
**Emotional expressivity**					
IG	1.065 (0.210)	1.084 (0.206)	1.156 (0.318)	1.450 (0.686)	1.399 (0.600)
CG	1.176 (0.366)	1.169 (0.285)	1.388 (0.474)	1.667 (0.588)	1.794 (0.792)
**Vocalizations**					
IG	1.244 (0.410)	1.069 (0.216)	1.165 (0.331)	1.365 (0.624)	1.522 (0.644)
CG	1.176 (0.366)	1.167 (0.365)	1.328 (0.480)	1.487 (0.510)	1.714 (0.800)

**Table 5:** M (SD) of the mood ratings at the respective measurement points.

	**M1**	**M2**	**M3**	**M4**
**Child**				
IG	1.794 (0.729)	1.483 (0.634)	1.781 (0.792)	
CG	1.800 (0.764)	2.042 (0.922)	2.304 (1.146)	
**Parent**				
IG	2.438 (0.982)	1.731 (0.724)	2.133 (0.681)	2.759 (1.154)
CG	2.440 (0.768)	2.498 (0.837)	2.487 (0.837)	3.333 (1.197)

**Table 6:** M (SD) of the PedsQL at the respective measurement points.

	**M1**			**M2**		
	**Total**	**Physical**	**Psychosocial**	**Total**	**Physical**	**Psychosocial**
**Child**						
IG	78.012 (12.500)	85.294 (10.840)	75.667 (14.469)	75.467 (12.291)	80.102 (16.636)	75.840 (12.951)
CG	72.241 (12.113)	81.929 (10.864)	72.179 (13.718)	79.698 (9.330)	83.681 (7.391)	78.037 (10.546)
**Parent**						
IG	75.070 (11.931)	80.855 (12.634)	73.386 (10.527)	76.045 (13.038)	80.425 (15.588)	75.747 (12.860)
CG	72.325 (12.214)	81.198 (10.571)	73.038 (10.943)	74.704 (10.545)	85.000 (8.398)	73.667 (14.845)

### mYPAS

#### Activity

There was a significant effect of time, *F*_(4, 137)_ = 3.456, *p* = 0.01, and *post-hoc* analyses showed that regardless of group, children had significantly higher activity scores at O5 (*p* = 0.015) compared to O1. Moreover, we found a significant fixed effect of CPRS conflict, *F*_(1, 39)_ = 5.086, *p* = 0.030, indicating that more reported conflicts were associated with higher mYPAS activity scores.

#### State of Apparent Arousal

For mYPAS arousal we found a significant fixed effect of time, *F*_(4, 137)_ = 3.336, *p* = 0.012, indicating that, regardless of group, children showed a trend toward significantly higher scores on arousal at O4 (*p* = 0.053) compared to O1. There was additionally a marginally significant fixed effect of CPRS conflict, *F*_(1, 39)_ = 3.585, *p* = 0.066, showing a positive association between arousal and CPRS conflict.

#### Emotional Expressivity

There was a marginally significant interaction between time and group, *F*_(4, 137)_ = 2.328, *p* = 0.059, for mYPAS emotions. Simple effects analyses showed that IG had significantly lower scores on mYPAS emotions than CG at O3 (β = 0.370, *SE* = 0.129, *p* = 0.005). There was also a significant fixed effect of time, *F*_(4, 137)_ = 6.276, *p* < 0.001, and *post-hoc* tests showed that children, regardless of group, had significantly higher scores at O4 (*p* = 0.014) and O5 (*p* = 0.023) than at O1. Moreover, we also found a significant positive association between mYPAS emotion scores and CPRS conflict, *F*_(1, 39)_ = 4.505, *p* = 0.040, suggesting that more reported relationship conflict goes along with more negative emotions during the clown visit.

#### Vocalizations

There was a significant interaction effect between group and time, *F*_(4, 137)_ = 3.499, *p* = 0.009, for mYPAS vocalizations, and follow-up analyses showed that the IG had significantly lower scores on mYPAS vocalizations than CG at O2 (β = 0.263, *SE* = 0.136, *p* = 0.055), O3 (β = 0.440, *SE* = 0.126, *p* = 0.001), and O4 (β = 0.396, *SE* = 0.196, *p* = 0.045).

### Mood Ratings

For children's self-reported mood ratings, we found a significant fixed effect of age, *F*_(1, 40)_ = 8.284, *p* = 0.006, showing that older children, in general, reported more negative mood (β = 0.011, *SE* = 0.004). There was also a fixed effect of CPRS closeness, *F*_(1, 40)_ = 4.047, *p* = 0.051, showing that children whose parents reported higher closeness in their relationship reported more negative mood (β = 0.043, *SE* = 0.026).

For parental mood ratings, there was a significant interaction between time and group, *F*_(3, 117)_ = 3.263, *p* = 0.024, and simple effects analyses showed that parents in IG reported significantly better mood (i.e., lower scores) than parents in CG at M2 (β = 0.790, *SE* = 0.275, *p* = 0.005) and M3 (β = 0.662, *SE* = 0.241, *p* = 0.007). Analyses also revealed significant interactions between group and CPRS closeness, *F*_(1, 40)_ = 6.581, *p* = 0.014, as well as CPRS conflict, *F*_(1, 40)_ = 5.812, *p* = 0.021. There was a positive association between parental mood reports and both CPRS closeness (β = 0.112, *SE* = 0.046, *p* = 0.020) and CPRS conflict (β = 0.080, *SE* = 0.033, *p* = 0.021) in CG, suggesting that parents in the control group who reported high closeness or conflict in their relationships with their children also reported more negative mood.

### PedsQL

#### Children's Self-Report

For total child-reported well-being, we found a marginally significant interaction between time and group, *F*_(1, 37)_ = 3.651, *p* = 0.064, and simple effects analyses showed that IG had significantly lower total well-being scores than CG at M2 (β = 7.623, *SE* β = 3.942, *p* = 0.061). There was also a marginally significant interaction between group and CPRS dependence, *F*_(1, 39)_ = 3.908, *p* = 0.055, indicating that there was a negative association between total well-being scores and CPRS dependence only in CG (β = −2.049, *SE* = 0.965, *p* = 0.040). While there were no significant effects of group, time or the relationship variables on child-reported physical well-being, there was a marginally significant interaction between time and group for psychosocial well-being, *F*_(1, 37)_ = 4.033, *p* = 0.052. Simple effects analyses showed that children in the intervention group reported significantly lower psychosocial well-being than children in the control group at time 2 (β = 8.602, *SE* = 4.241, *p* = 0.049).

#### Parental Proxy-Report

For parent-reported total well-being, there was a significant fixed effect of CPRS conflict, *F*_(1, 40)_ = 9.729, *p* = 0.003, indicating a negative association between parent-reported total well-being scores and CPRS conflict for all children in the sample (β = −1.127, *SE* = 0.383). Similar to children's reports, we found no significant effects for parent-reported physical well-being. For parent-reported psychosocial well-being, there was also a significant fixed effect of CPRS conflict, *F*_(1, 40)_ =11.113, *p* = 0.002, indicating a negative association between parent-reported total well-being scores and CPRS conflict for all children in the sample (β = −1.093, *SE* = 0.396).

### Correlations Between Outcome Measures

All correlation matrices are provided as Supplementary Tables. Correlational analyses between mYPAS and children's mood ratings (see Supplementary Table 1) showed only few significant and unsystematic relationships. For IG, there was a significant relationship between children's mood at M1 and mYPAS arousal at O4, as well as between children's mood at M3 and mYPAS vocalizations at O2. For CG, children's mood at M1 was correlated with mYPAS emotions at O1 and emotions and arousal at O5. Moreover, children's mood at M2 was significantly related to mYPAS arousal, emotions and vocalizations at O1, as well as to mYPAS emotions at O2.

Analyses between mYPAS and children's self-reported HRQL (see Supplementary Table 2) revealed only one significant negative correlation between these two measures in IG, specifically between mYPAS arousal at O1 and physical well-being at M1. For CG, physical well-being at M1 was negatively correlated with mYPAS emotions and vocalizations at O1, and also with mYPAS emotions at O4 and O5. Total child-reported well-being at M1 was also found negatively correlated with mYPAS emotions at O5. In contrast, psychosocial and total self-reported well-being at M1 was positively correlated with mYPAS vocalizations at O2. Similarly, physical child-reported well-being at M2 was negatively correlated with mYPAS activity and arousal at O4, while psychosocial and total well-being at M2 were positively related to mYPAS activity at O2. Finally, analyses between children's mood reports and their self-reported HRQL (see Supplementary Table 3) revealed only a significant correlation between mood and psychosocial well-being at M2 for IG.

Additionally, we were interested in the relationship between children's and parental mood ratings in the two groups (see Supplementary Table 4). Interestingly, while in IG there were no significant correlations between parental and children's mood ratings, for CG we found that mood ratings of children and parents are aligned, especially at M2 and M3. Moreover, in this group parental mood at M1 was also significantly correlated with children's mood rating at M2 and M3.

Finally, we examined the correspondence between child self-reported and parental proxy-reported well-being, and the detailed results are displayed in Supplementary Table 5. For IG at M1 there were only two significant correlations between self-reported and parental-reported children's well-being, while at M2 most of the correlations between these two reports reached the significance level. In contrast, for CG there were only three significant correlations at M1 and two at M2 between self-reported and proxy-reported children's well-being.

## Discussion

In recent years, it has been argued that humor and laughter have unique properties, which can help in coping with pain and stress (8). While a healthcare clown intervention was found successful in reducing children's crying, diminishing pain and anxiety, as well as postoperative maladaptive behavior (15, 17, 20, 22, 77), little research has, thus far, examined whether healthcare clowns also have a positive influence on the emotional well-being of pediatric patients and their relatives. Consequently, in the present study we have examined whether a healthcare clown intervention evokes positive emotions and improves well-being in hospitalized children and their parents, and assessed the role of child-parent relationship aspects on this intervention effect.

We observed children at different stages of preparation for a non-emergent surgery and used mYPAS to rate their activity, arousal, emotions, and vocalizations. Our results showed that children in the intervention group vocalized more positively than children in the control group during the waiting, premedication, and bed manipulation phase (i.e., O2, O3, and O4, respectively), and also showed more positive emotions than children in the control group during premedication (i.e., O3). It is interesting to note here that differences between the groups were not evident during the familiarization phase, where children in the intervention group had the longest undistracted interactions with the clowns. Instead, the differences appeared during situations, where the clowns were “just” another person in the room, without having the sole attention of the children. It could be argued that especially during premedication and bed manipulation children realized the seriousness of the situation and this was when the clowns were effective in evoking more positive behaviors in children. This interpretation would jibe with our finding that children in both groups were rated as showing more negative activity during bed manipulation (i.e., O4) and higher arousal on the way to the operating room (i.e., O5). It seems that these observation points, which were just ahead of the surgical procedure, were particularly and equally distressing to all children. Even though we did not find an effect of the clown intervention on children's activity or arousal, children in the intervention group displayed more positive behaviors, especially in situations leading up to the actual way to the OR, showing some, albeit limited effectiveness of healthcare clowns.

Interestingly, we found a positive effect of the clown intervention on children's behavior during the waiting phase when the clowns were not present in the room. This suggests a possible carry-over effect of the clown intervention on to the interaction between children and their caregivers when they were alone. One explanation could be that children and their parents were talking about the clowns during their absence, which would explain why we found this effect for children's vocalizations as assessed on the mYPAS. Alternatively, it could be argued that, in line with our hypothesis, the clowns improved the mood within the dyads and this positive emotionality was not just momentary but had some longer-lasting effects. However, the fact that we did not find any differences between the groups on children's emotionality during the waiting phase as rated on the mYPAS weakens this interpretation. It is possible that children felt no imminent fear or anxiety during the waiting phase, as nothing was happening at this point in time. Moreover, parents in the intervention group reported significantly better mood than parents in the control group, and this was the case particularly during the waiting and the premedication phases. These results suggest that the clown intervention not only directly improved parental mood during a possibly stressful event for their children (i.e., administration of unenjoyable premedication drops), but also indirectly by allowing parents to feel more uplifted even when the clowns were not present. Thus, parental mood could have subsequently affected their children's behaviors.

In contrast with our hypotheses and the findings on the mYPAS, we found that children's self-reported mood was unaffected by the clown intervention. While these results jibe with some previous research [e.g., (34)], it could be that in the present study the divergent findings were a function of the assessment method and, in fact, we found only few correlations between these two measures. Specifically, the mYPAS was evaluated by trained observers while children were asked to rate their own mood, which requires a certain level of introspection [see e.g., (78)]. That is, children had to perceive their own feelings, and explicitly report them, even if in a non-verbal way, which could have been too difficult for some children in our sample. Our finding that older children reported more negative mood regardless of group fits with this interpretation. Only a few previous studies examining children's emotions in a hospital setting reported age effects, and Glazebrook et al. (79), for example, found that younger children showed more distress during anesthesia induction. However, in their study children's distress was rated by independent observers. Thus, it could be argued that older children not only had the necessary introspection level, but also may have been more aware of the hospital context and the upcoming procedure, and this made them prone to report more negative mood. Consequently, the mYPAS seems to better reflect children's social and emotional behavior across the wide age range examined in the present study.

Our results further showed that while both groups of children reported similar levels of HRQL before commencement of the study, children in the control group reported higher total and especially psychosocial well-being at the second measurement point (i.e., at the end of the waiting period right before premedication) than children in the intervention group. However, this was not reflected in parent-reported well-being of their children, suggesting that it may be a child-specific effect. In line with this interpretation, we found only two (of nine) significant correlations between children's and parental reports on the PedsQL in the control group at the second measurement point (in contrast, six of nine correlations between children's and parental PedsQL reports in the intervention group reached significance). Moreover, we found only few and unsystematic correlations between PedsQL and other utilized measures (i.e., mYPAS, mood ratings), suggesting that this was a specific effect pertaining to children's reports of their subjective perception of the impact of their health status, including disease, and treatment, on physical, psychological, and social functioning [see (80), for this definition of HRQL].

It is possible that close to the surgery children in the control group focused more on getting better after the surgery rather than on their momentary feelings, and this could be a function of the effect of parental presence. Previous research showed that parental presence is associated with lower anxiety in children undergoing surgery [e.g., (6)], and thus could potentially increase children's experienced well-being. Parental presence during medical procedures has various reported benefits, such as decreasing separation anxiety [e.g., (81)], increasing child cooperation [e.g., (82)], and enhancing parental satisfaction [e.g., (82–84)]. Interestingly, Caldwell-Andrews et al. (85) found that children of mothers who were highly motivated to be present during anesthetic induction were more anxious than children of mothers who were less motivated to enter the OR. These mothers also reported higher state anxiety during anesthetic induction. The authors suggested that (a) some anxious mothers have a high desire to be present during anesthetic induction in order to manage their own anxiety, and their anxiety may, in turn, elevate their children's anxiety, while (b) some mothers may have less desire to be present in the OR as a function of their confidence in their child's ability to cope with the experience (85). In our data, we also found that when parents reported higher dependency in their relationship with children, children in the control group reported lower well-being, suggesting that when parents perceived their children as more independent, then children's HRQL score was higher. These results indicate that parental own emotional and relationship experiences may have had a particular impact on children in the control group.

Parental mood in the control group was also linked with their reported relationship qualities; specifically, parents in the control group who reported more closeness as well as conflicts with their children also reported more negative mood in general. While we have expected that more positive relationship aspects, such as closeness, are generally associated with positive emotions and mood, it is possible that the closer parents felt to their children the more concerned they might have been for their health and safety which could have affected their mood. Eventually, this concern was likely transferred to their children, because we found that children, regardless of group, reported more negative mood when parents felt closer to them. The finding that parents reporting conflictual relationships with their children appeared to be in a more negative mood is more aligned with previous literature and thus our hypotheses. In potentially stressful situations, such as waiting for a surgery, conflicts could have arisen for those dyads who reported to have more conflicts in general in their relationship, which could then have affected parental mood. We can only speculate whether a healthcare clown intervention could have buffered these effects, especially because we did not find the reverse pattern in the intervention group, i.e., that parents in the intervention group reported better mood depending on the relationship qualities. However, given the finding that the clown intervention improved parental mood, we could argue that clowns exerted a positive effect on parents, and this could have minimized the impact of negative relationship aspects. In turn, in the control group, where children and parents experienced no distraction by clowns, the focus was solely on the dyad which could have exacerbated these effects. In fact, our analyses showed that mood ratings of children and their parents in the control group were more aligned, especially at the second and third measurement point, which was not the case in the intervention group. Existing research would support this interpretation by showing that child anxiety and distress during anesthetic induction is associated with parents' level of anxiety [e.g., (79, 86–88)] suggesting that parental and children's emotional feelings are coordinated especially during stressful events.

We also found that when parents reported more conflicts in the relationship with their children, then children in both groups showed more negative behaviors as rated on the mYPAS (i.e., activity, emotions, arousal). This finding jibes with research showing that parental presence may also have negative implications on the pre-surgery routine, including elevation of parental anxiety, disruption of the routine and increase in child behavioral problems [see (6), for review]. Similarly, parental presence in dyads reporting more relationship conflicts could have had adverse effects on children's behaviors, regardless of group, albeit for different reasons. As previously argued, in the control group the presence of the parent and lack of a distraction (e.g., through the clown) could have exacerbated the negative relationship effects in that the dyads could have experienced more conflicts in this stressful situation. On the other hand, it could be argued that children who previously experienced more conflict with their parents also reacted more negatively to the clown intervention, which would be in line with the previously discussed findings of McDowell et al. (67). It is possible that these children had an increased sense of helplessness which affected the activation of their regulation strategies. Subsequent research needs to study in more detail which aspects of parent-child relationships play a relevant role for assessing the effectiveness of the healthcare clown intervention.

There are several limitations of the current study that may have had an impact on our findings. First, we tested a relatively small sample and thus the current results need to be replicated with larger, more representative groups. Even though our sample was rather heterogeneous in terms of its socio-economic characteristics, it was still skewed toward those families that likely value the work of healthcare clowns in the first place. Thus, our positive results in terms of the effectiveness of this intervention may be explained by this factor. Second, we have tested children within a relatively wide age range and thus we cannot rule out developmental differences in humor understanding and appreciation as a potential explanation of our results. Nonetheless, we found very few effects of age in our analyses. It should be noted that healthcare clowns are trained and especially sensitive to the capabilities of their young audience, and so we could argue that they appropriately adjusted their play according to children's developmental levels. A related and a potential third limitation is that the experiences of children with the clowns in the intervention group varied, because clowns did not follow a standardized clown play script. We were cautious to leave clowns their artistic freedom, while we realize that this may have introduced substantial variations in their actions. However, healthcare clowns do not follow standardized behavioral scripts in their everyday work, and thus restricting their range of actions would not have been representative of their work and lack validity. Finally, we need to consider potential drawbacks of some of the measures we have utilized in this study. We would have benefited from a more direct assessment of the emotions the clown intervention evoked by asking parents and children to characterize them in more detail [see e.g., (32)]. While this would have been quite challenging in the present context considering the developmental differences of children between 5 and 12 years of age, future research needs to find more detailed ways to measure the emotions experienced by children during a healthcare clown intervention. Moreover, our measure of parent-child relationships was based solely on parental reports and we did not differentiate between reports of mother, fathers, and other caregivers, which could have thwarted our results. Yet, because this is the first study that considered the role of parent-child relationships, we are just beginning to understand which relationship aspects may play a particularly important role. Future studies utilizing observational data as well as children's reports need to examine more general aspects of parent-child relationships, such as e.g., sensitivity, as well as more specific aspects related to stressful situations, such as e.g., emotion regulation strategies or social referencing, to find out what exactly about parent-child relationship it is that may influence the effectiveness of the healthcare clown intervention.

Despite these limitations, results of the present study demonstrate that a healthcare clown intervention has some positive effects on behaviors and mood of hospitalized children and their parents, which could further lead to more compliance and faster recovery. Our findings also suggest that we need to consider the pre-existing “relationship microcosmos” that the clowns enter when assessing their effectiveness as a hospital intervention. Specifically, we could show that parent-child relationship variables play an indisputable role for the establishment of a relationship with the clowns which may, subsequently, allow for the emergence of positive emotionality and well-being in a frightening hospital context. This idea, of course, is not a new one - attachment theoretical arguments suggest that the relationship with a primary caregiver is formative for any subsequent relationships. In this study we provided first evidence that this may even apply for very short-lived relationships, such as between children and healthcare clowns. Furthermore, findings of our study are in line with the healthcare clowns' general non-clown-centric attitude. That is, clowns, by default, direct their attention at everybody who watches. Thus, the hypothesis that the mood of one person is in direct relationship to the mood of all the others is an intrinsic part of the clown work. However, despite the positive effects of the healthcare clown intervention on parents, the focus of clowns should remain with the children, as parents probably experience positive emotions precisely because they perceive their children as being at ease. Future research must dig deeper into the workings of the child-caregiver dyad to investigate in more detail the specific factors that not only impact the effectiveness of the work of healthcare clowns, but also could be amended by an intervention through humor.

## Data Availability Statement

The raw data supporting the conclusions of this article will be made available by the authors, without undue reservation.

## Ethics Statement

The study involved human participants and was reviewed and approved by the Ethics committee of the Motol University Hospital, Prague, Czechia. Written informed consent to participate in this study was provided by the participants' legal guardian/next of kin.

## Author Contributions

GM, LH, and ZK conceptualized the study. LH and ZK supervised data collection. GM analyzed the data and wrote the paper. All authors contributed to the article and approved the submitted version.

## Conflict of Interest

The authors declare that the research was conducted in the absence of any commercial or financial relationships that could be construed as a potential conflict of interest.
