# Supplementary material for: To the Operating Room! Positive Effects of a Healthcare Clown Intervention on Children Undergoing Surgery
Source: Front Public Health. 2021 Apr 20;9:653884. doi: 10.3389/fpubh.2021.653884 (PMC8093515; doi:10.3389/fpubh.2021.653884)
Supplement: Supplementary file 1 [file Table_1.DOCX]

**sTable 1**

*Correlational analyses between mYPAS and children’s mood ratings*

|  | **M1 mood** | **M2 mood** | **M3 mood** |
| --- | --- | --- | --- |
|  | ***IG*** | | |
| **O1 Activity** | .088 | .172 | .105 |
| **Arousal** | .280 | .219 | .134 |
| **Emotions** | .223 | .222 | .136 |
| **Vocalizations** | .074 | .145 | .100 |
| **O2 Activity** | .067 | .032 | .321 |
| **Arousal** | .067 | .032 | .321 |
| **Emotions** | .047 | .036 | .323 |
| **Vocalizations** | .056 | -.019 | **.429*** |
| **O3 Activity** | .224 | .176 | .095 |
| **Arousal** | .212 | .198 | .035 |
| **Emotions** | .298 | .102 | .031 |
| **Vocalizations** | .065 | .060 | -.073 |
| **O4 Activity** | .451† | .035 | .145 |
| **Arousal** | **.490*** | .036 | .034 |
| **Emotions** | .397 | -.106 | .039 |
| **Vocalizations** | .427 | -.085 | .168 |
| **O5 Activity** | .214 | .010 | .257 |
| **Arousal** | .381 | .051 | .166 |
| **Emotions** | .351 | .087 | .118 |
| **Vocalizations** | .313 | -.028 | .167 |
|  | ***CG*** | | |
| **O1 Activity** | .052 | .149 | -.007 |
| **Arousal** | .325 | **.405*** | .003 |
| **Emotions** | **.451*** | **.528**** | .098 |
| **Vocalizations** | .384 | **.468*** | .062 |
| **O2 Activity** | -.122 | -.104 | .184 |
| **Arousal** | -.029 | .039 | .243 |
| **Emotions** | .036 | .069 | .336 |
| **Vocalizations** | -.131 | -.046 | .112 |
| **O3 Activity** | .081 | -.361 | -.110 |
| **Arousal** | .489 | -.141 | -.006 |
| **Emotions** | .232 | -.219 | -.039 |
| **Vocalizations** | .046 | -.097 | .044 |
| **O4 Activity** | .110 | .123 | .023 |
| **Arousal** | .110 | .123 | .023 |
| **Emotions** | .154 | .389 | -.077 |
| **Vocalizations** | .261 | .163 | .154 |
| **O5 Activity** | .065 | .016 | -.001 |
| **Arousal** | **.475*** | .332 | -.039 |
| **Emotions** | **.466*** | **.485*** | .036 |
| **Vocalizations** | .405 | .308 | .057 |

†*p* < .06. **p* < .05. ***p* < .01. ****p* < .001.
