# Supplementary material for: To the Operating Room! Positive Effects of a Healthcare Clown Intervention on Children Undergoing Surgery
Source: Front Public Health. 2021 Apr 20;9:653884. doi: 10.3389/fpubh.2021.653884 (PMC8093515; doi:10.3389/fpubh.2021.653884)
Supplement: Supplementary file 2 [file Table_2.DOCX]

**sTable 2**

*Correlational analyses between mYPAS and children’s self-reported HRQL*

|  | **M1** | | | **M2** | | |
| --- | --- | --- | --- | --- | --- | --- |
|  | **physical** | **psycho-social** | **total** | **physical** | **psycho-social** | **total** |
|  | ***IG*** | | | | | |
| **O1 Activity** | -.056 | -.025 | -.020 | .178 | .235 | .270 |
| **Arousal** | **-.408*** | -.046 | -.152 | -.163 | .191 | .062 |
| **Emotions** | -.292 | -.031 | -.101 | -.035 | .219 | .151 |
| **Vocalizations** | -.214 | .184 | .024 | -.044 | .184 | .067 |
| **O2 Activity** | -.045 | -.140 | -.278 | .248 | -.059 | -.062 |
| **Arousal** | -.045 | -.140 | -.278 | .248 | -.059 | -.062 |
| **Emotions** | -.028 | -.121 | -.249 | .240 | -.073 | -.064 |
| **Vocalizations** | -.049 | -.218 | -.153 | .265 | -.022 | .173 |
| **O3 Activity** | -.088 | .075 | .030 | .097 | .107 | .132 |
| **Arousal** | -.090 | .112 | -.106 | .119 | .061 | -.045 |
| **Emotions** | -.134 | .077 | -.020 | .079 | .106 | .090 |
| **Vocalizations** | .039 | .294 | .105 | .124 | .167 | .062 |
| **O4 Activity** | -.392 | -.224 | -.223 | .060 | .182 | .225 |
| **Arousal** | -.285 | -.174 | -.324 | .083 | .083 | .011 |
| **Emotions** | -.254 | -.281 | -.201 | -.002 | .186 | .193 |
| **Vocalizations** | -.163 | -.194 | -.119 | .129 | .008 | .157 |
| **O5 Activity** | .048 | -.050 | .042 | .376 | .137 | .324 |
| **Arousal** | -.027 | -.032 | -.057 | .236 | .161 | .190 |
| **Emotions** | -.045 | -.011 | -.098 | .157 | .219 | .130 |
| **Vocalizations** | -.002 | -.060 | -.141 | .167 | -.034 | -.042 |
|  | ***CG*** | | | | | |
| **O1 Activity** | -.303 | .252 | -.202 | .185 | .292 | .295 |
| **Arousal** | -.306 | .231 | -.206 | -.179 | .025 | -.026 |
| **Emotions** | **-.426*** | .164 | -.228 | -.239 | -.025 | -.084 |
| **Vocalizations** | **-.454*** | .224 | -.193 | -.084 | .121 | .080 |
| **O2 Activity** | -.044 | .277 | .254 | .250 | **.531*** | **.535*** |
| **Arousal** | .153 | .254 | .307 | .313 | .481 | .506† |
| **Emotions** | .067 | .152 | .243 | .169 | .304 | .313 |
| **Vocalizations** | .273 | **.479*** | **.513*** | .233 | .502† | .505† |
| **O3 Activity** | -.122 | -.084 | .009 | -.355 | -.140 | -.193 |
| **Arousal** | -.099 | -.207 | -.101 | -.247 | -.384 | -.391 |
| **Emotions** | -.511 | -.088 | -.053 | -.208 | -.076 | -.108 |
| **Vocalizations** | -.454 | -.209 | -.152 | -.277 | -.160 | -.195 |
| **O4 Activity** | -.269 | -.154 | -.230 | **-.652*** | -.052 | -.308 |
| **Arousal** | -.269 | -.154 | -.230 | **-.652*** | -.052 | -.308 |
| **Emotions** | **-.698**** | .131 | -.168 | -.158 | .328 | .207 |
| **Vocalizations** | -.372 | -.061 | -.195 | .041 | .084 | .086 |
| **O5 Activity** | -.356 | -.139 | -.350 | -.089 | -.060 | -.073 |
| **Arousal** | -.316 | -.070 | -.279 | -.290 | -.146 | -.196 |
| **Emotions** | **-.683**** | -.142 | **-.493*** | -.193 | -.126 | -.154 |
| **Vocalizations** | -.387 | -.112 | -.276 | -.065 | -.123 | -.119 |

†*p* < .06. **p* < .05. ***p* < .01. ****p* < .001.
