# Supplementary material for: To the Operating Room! Positive Effects of a Healthcare Clown Intervention on Children Undergoing Surgery
Source: Front Public Health. 2021 Apr 20;9:653884. doi: 10.3389/fpubh.2021.653884 (PMC8093515; doi:10.3389/fpubh.2021.653884)
Supplement: Supplementary file 3 [file Table_3.DOCX]

**sTable 3**

*Correlational analyses between children’s mood reports and their self-reported HRQL*

|  | **M1** | | | **M2** | | |
| --- | --- | --- | --- | --- | --- | --- |
|  | **physical** | **psycho-social** | **total** | **physical** | **psycho-social** | **total** |
|  | ***IG*** | | | | | |
| **M1 mood** | -.286 | -.149 | -.229 | .239 | .269 | .288 |
| **M2 mood** | -.030 | -.029 | -.106 | .085 | **.514*** | .306 |
| **M3 mood** | -.166 | -.134 | -.078 | .407† | -.060 | .263 |
|  | ***CG*** | | | | | |
| **M1 mood** | -.269 | -.328 | -.145 | -.376 | -.299 | -.342 |
| **M2 mood** | -.428† | -.361 | -.365 | -.419 | -.343 | -.390 |
| **M3 mood** | .026 | -.239 | -.086 | -.063 | .064 | .042 |

†*p* < .06. **p* < .05. ***p* < .01. ****p* < .001.
