# Supplementary material for: To the Operating Room! Positive Effects of a Healthcare Clown Intervention on Children Undergoing Surgery
Source: Front Public Health. 2021 Apr 20;9:653884. doi: 10.3389/fpubh.2021.653884 (PMC8093515; doi:10.3389/fpubh.2021.653884)
Supplement: Supplementary file 4 [file Table_4.DOCX]

**sTable 4**

*Correlational analyses between children’s and parental mood ratings in the two groups*

|  | **M1**  **parental mood** | **M2**  **parental mood** | **M3**  **parental mood** | **M4**  **parental mood** |
| --- | --- | --- | --- | --- |
|  | ***IG*** | | | |
| **M1 child mood** | -.268 | -.127 | .125 | .092 |
| **M2 child mood** | .225 | -.190 | -.097 | -.098 |
| **M3 child mood** | .132 | .072 | .162 | -.116 |
|  | ***CG*** | | | |
| **M1 child mood** | .304 | .356 | -.144 | -.061 |
| **M2 child mood** | **.438*** | **.434*** | .184 | .173 |
| **M3 child mood** | **.429*** | .355 | **.584**** | .071 |

†*p* < .06. **p* < .05. ***p* < .01. ****p* < .001.
