# Supplementary material for: To the Operating Room! Positive Effects of a Healthcare Clown Intervention on Children Undergoing Surgery
Source: Front Public Health. 2021 Apr 20;9:653884. doi: 10.3389/fpubh.2021.653884 (PMC8093515; doi:10.3389/fpubh.2021.653884)
Supplement: Supplementary file 5 [file Table_5.DOCX]

**sTable 5**

*Correlational analyses between child self-reported and parental proxy-reported HRQL*

|  | **Child** | | | | | |
| --- | --- | --- | --- | --- | --- | --- |
|  | **M1 physical** | **M1 psycho-social** | **M1**  **total** | **M2 physical** | **M2 psycho-social** | **M2**  **total** |
| **Parental** | ***IG*** | | | | | |
| **M1 physical** | .179 | .042 | .086 | **.574**** | **.667***** | **.808***** |
| **M1 psychosocial** | .089 | **.421*** | .327 | .101 | **.840***** | **.607**** |
| **M1 total** | .138 | .223 | **.385*** | .321 | **.795***** | **.860***** |
| **M2 physical** | **.444*** | -.025 | .159 | **.731***** | .333 | **.675***** |
| **M2 psychosocial** | -.024 | .332 | .236 | .036 | **.853***** | **.601**** |
| **M2 total** | .223 | .139 | .386† | .425† | **.716***** | **.867***** |
|  | ***CG*** | | | | | |
| **M1 physical** | **.560**** | .168 | .352 | **.706**** | .000 | .173 |
| **M1 psychosocial** | .181 | **.462*** | .261 | .158 | **.526*** | **.497*** |
| **M1 total** | .361 | .141 | **.677***** | .161 | .149 | .169 |
| **M2 physical** | .385 | -.054 | .097 | .290 | -.210 | -.102 |
| **M2 psychosocial** | .056 | **.490*** | .098 | .360 | **.668*** | **.659*** |
| **M2 total** | .185 | .175 | **.551*** | .284 | .422 | .431 |
